# Didelphys Uterus: A Case Report and Review of the Literature

**DOI:** 10.1155/2015/865821

**Published:** 2015-09-07

**Authors:** Shadi Rezai, Pameela Bisram, Isamarie Lora Alcantara, Ruchi Upadhyay, Carla Lara, Malvina Elmadjian

**Affiliations:** ^1^Department of Obstetrics and Gynecology, Lincoln Medical and Mental Health Center, Bronx, NY 10451, USA; ^2^St. George's University, School of Medicine, St. George's, Grenada

## Abstract

*Background*. Mullerian duct anomalies (MDAs) are congenital defects of the female genital system that arise from abnormal embryological development of the Mullerian ducts. A didelphys uterus, also known as a “double uterus,” is one of the least common amongst MDAs. This report discusses a case of didelphys uterus that successfully conceived, carried her pregnancy to term, and delivered vaginally without any significant complications. *Case*. Patient is a 29-year-old G2P0010 from Bangladesh, initially came a year prior in her first pregnancy, with spontaneous abortion (SAB). Pelvic Sonogram at that time showed a diagnosis of bicornuate versus didelphys uterus. There were no renal anomalies on subsequent abdominal CT scan. Patient presented with the second pregnancy and had uncomplicated prenatal care and did not have signs of preterm labor; fetus showed appropriate growth and the pregnancy was carried in the left uterus. Patient presented at 38 4/7 wks with Premature Rupture of Membrane and underwent induction of labor with Cytotec. Antibiotics were started for chorioamnionitis. Patient had a vaginal delivery with left mediolateral episiotomy and complete tear of vaginal septum. Third stage of labor was complicated with retained placenta, which was removed manually in the operating room with total EBL of 600 cc.

## 1. Introduction

Mullerian duct anomalies (MDAs) are congenital defects of the female genital system that arise from abnormal embryological development of the Mullerian ducts. These abnormalities can include failure of development, fusion, canalization, or reabsorption, which normally occurs between 6 and 22 weeks in utero. Most sources estimate an incidence of these abnormalities to be from 0.5 to 5.0% in the general population [1–4].

Septate uterus is the commonest uterine anomaly with a mean incidence of ~35% followed by bicornuate uterus (~25%) and arcuate uterus (~20%) [4]. Uterine anomalies may have a part in the delayed natural conception of women with mainly secondary infertility [4].

It is generally accepted that having a uterine anomaly is associated with poorer pregnancy outcomes such as increased chances of spontaneous abortion, premature labor, cesarean delivery due to breech presentation, and decreased live births, compared to a normal uterus [1–5]. However, the degree of these outcomes varies among different types of uterine anomalies.

Unicornuate and didelphys uterus have term delivery rates of ~45%, and the pregnancy outcome of patients with untreated bicornuate and septate uterus is also poor with term delivery rates of only ~40% [4]. Arcuate uterus is associated with a slightly better but still impaired pregnancy outcome with term delivery rates of ~65% [4].

Most women with a didelphys uterus are asymptomatic, but some present with dyspareunia or dysmenorrhea in the presence of a varying degree of longitudinal vaginal septum. Rarely, genital neoplasms, hematocolpos/hematometrocolpos, and renal anomalies are reported in association with didelphys uterus. Despite some of these complications, there are many cases of women with a didelphys uterus that did not exhibit any reproductive or gestational challenges.

When classifying these anomalies solely based on abnormal development, four major types are apparent:

1. Complete or partial failure of Mullerian duct development (agenesis; unicornuate uterus without a rudimentary horn);
2. Failure of ducts to canalize (unicornuate uterus with a rudimentary horn without proper cavities);
3. Incomplete fusion of Mullerian ducts (bicornuate or didelphys uterus) [6, 7];
4. Incomplete reabsorption of uterine septum (septate or arcuate uterus) [4].

The most recent and widely used classification systems for the different types of Mullerian duct abnormalities were created by Buttram Jr. and Gibbons (1979) [8, 9] and the American Fertility Society (1988) [3, 10, 11].

The modalities for correct diagnosis frequently used include highly invasive methods such as hysteroscopy, hysterosalpingography, and laparoscopy/laparotomy. However, these methods rely on the clinician's subjective interpretation rather than strict diagnostic criteria [10]. A 2D ultrasound is usually the first type of imaging done; however it is inadequate for diagnosis as it cannot reliably differentiate between subtypes of MDAs. The use of 3D ultrasound is becoming more commonly used for diagnosis as it is not only noninvasive, but it also overcomes the limitation of 2D ultrasound by providing a coronal view that enables examination of both the endometrial cavity and uterine fundus, thus giving all the information needed for morphological classification [10, 12]. Magnetic resonance imaging is also just as accurate and valuable in diagnosing MDAs as hysterosalpingograms, hysteroscopy, and laparoscopy are, even more so as it is noninvasive and can diagnose associated urinary tract abnormalities at the same time [13]. Nonetheless, it is still difficult to distinguish between these different anomalies on imaging modalities due to subjectivity; differences in morphology are often subtle and changing classification systems [4]. Despite these difficulties, a review of the prevalence of different types of uterine malformations done by Grimbizis et al. revealed that the septate uterus is most common at 35% followed by bicornuate at 25%, then arcuate at 20%, then unicornuate at 9.6%, and complete agenesis at 3%. Didelphys uterus was found to be the second least common at 8.3% of all MDAs [4].

A didelphys uterus is characterized by complete failure of the Mullerian ducts to fuse leading to separate uterine cavities and two cervices. A longitudinal vaginal septum is also present that may range from thin and easily displaced to thick and inelastic. Initial suspicion of the condition followed by the diagnosis usually begins with a routine speculum exam where visualization of anatomical abnormalities warrants further investigation. Further, because the Mullerian ducts develop often in association with Wolffian ducts, abnormalities of the kidneys may be found in conjunction with uterine abnormalities [1, 2].

In this case report, we discuss a rare case of didelphys uterus in a women with a history of spontaneous abortion who successfully conceived, carried her pregnancy to term, and delivered vaginally without any complications.

## 2. Presentation of Case

**Figure 1:**
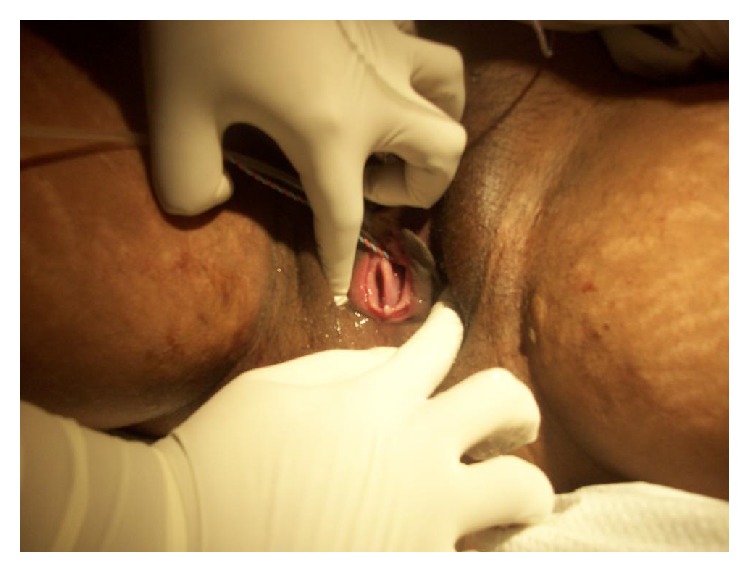
Noncommunicable vaginal septum

This patient is a 29-year-old G2P0010 from Bangladesh, who initially came a year before in her first pregnancy, with spontaneous abortion (SAB). Pelvic sonogram at that time showed a diagnosis of bicornuate versus didelphys uterus. On exam, patient had a noncommunicating, thick vaginal septum (Figure 1); however patient and her husband were not aware of the patient condition until that day. There were no renal anomalies on subsequent abdominal CT scan. The patient did not report having dyspareunia, dysmenorrhea, or chronic abdominal pain in the past.

Patient presented with the second pregnancy, which was seen and evaluated by the general gynecologist and diagnosis of didelphys uterus was confirmed. Patient was seen by Maternal Fetal Medicine (MFM) with suggestion for routine prenatal care at Ob-high risk clinic due to this uterine anomaly and its associated risks.

Patient had uncomplicated prenatal care and did not have any signs of bleeding or threatened preterm labor, her labs and vital signs remained within normal limits, and fetal ultrasounds/tracings also remained within normal limits. Fetus showed appropriate growth and the pregnancy was carried in the left uterus.

**Figure 2:**
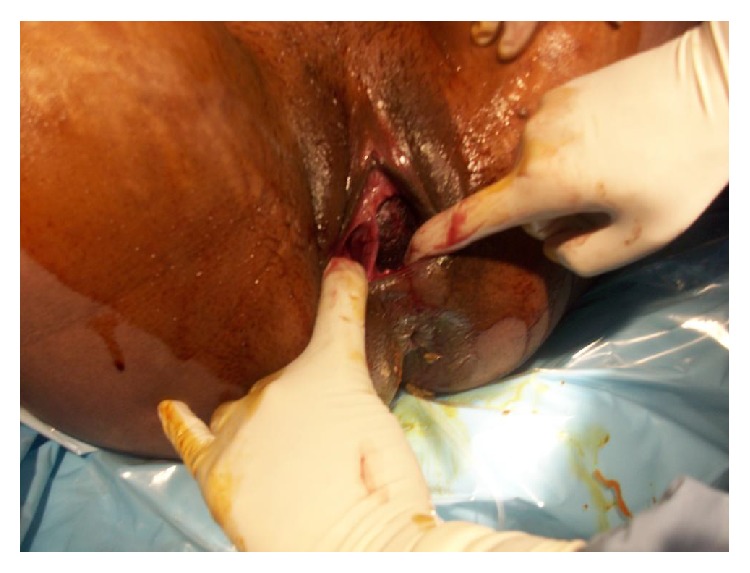
More tear of vaginal septum as patient continues to push and fetal head descends down.

At 38 weeks and 4 days, the patient presented with sudden vaginal leakage of clear fluids with some bloody mucoid discharge. On physical exam she was not in any distress, normotensive, and afebrile. Gross pooling of clear fluid was found on speculum exam and the left cervix was 1/50/−3 with the right cervix closed. The fetal heart tracing was category one, showing a fetal heart rate of 150 at baseline, moderate variability, with accelerations and no decelerations. The bedside sonogram at that time showed the fetus to be in vertex position, placenta positioned anteriorly. At the end of this work-up, the patient was admitted for Premature Rupture of Membranes (PROM) and underwent induction of labor with Cytotec. Penicillin was started due to GBS positive status. Pain was controlled by epidural anesthesia. Due to protracted labor, Pitocin augmentation was indicated and patient progressed to second stage of labor. At full cervical dilatation of the left cervix, the right cervix was managed to be measured and was also 4-5 cm dilated. Vaginal septum started to tear with descent of fetal head and maternal pushing through the process of delivery (Figure 2).

Patient had a vaginal delivery of a baby boy with weight of 2660 grams with left mediolateral episiotomy and complete tear of vaginal septum.

**Figure 3:**
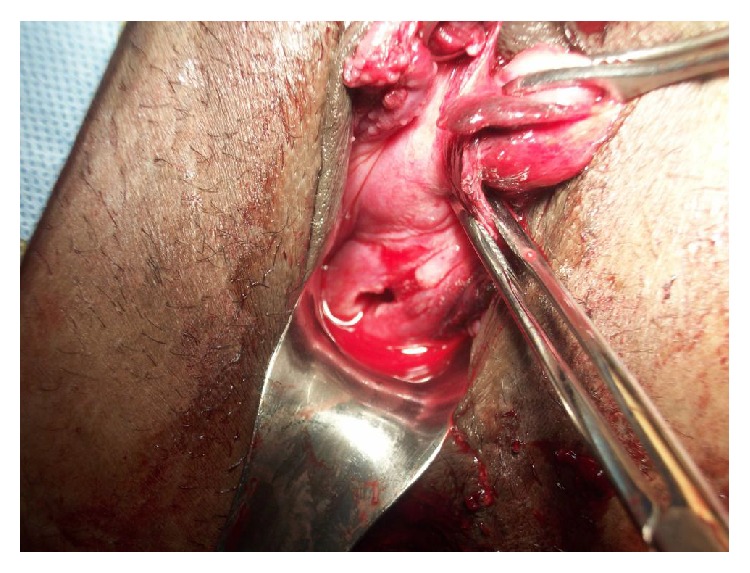
Patient was taken to operating room for removal of retained placenta; 2 separated cervices at 2 o'clock (postpartum cervix) and 7 o'clock (nonpregnant cervix) as well as completely torn vaginal septum (at 11 o'clock) are shown. Note that vaginal septum was completely destructed as fetal head delivered.

Patient had retained placenta, which was removed manually in the operating room with total EBL of 600 cc. Picture from the operating room shows two cervices next to each other (Figure 3).

**Figure 4:**
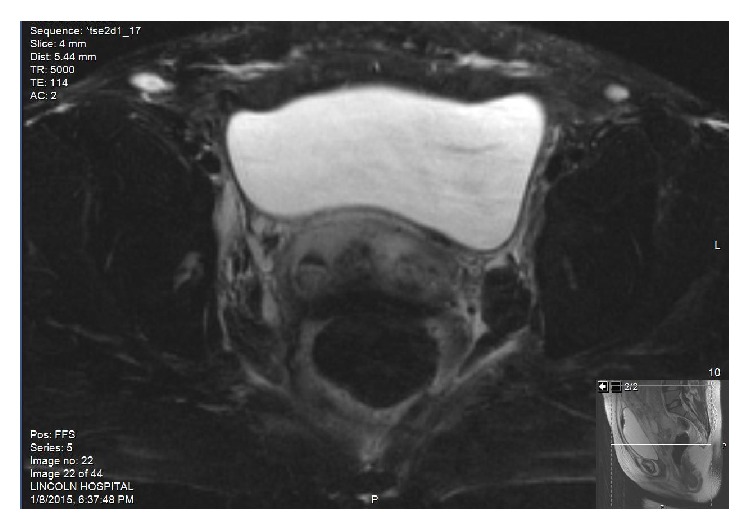
MRI of abdomen and pelvis with contrast: Series # 5, T2 axial FS: one cervix on the right and one cervix on the left, 2 separate cervices.

**Figure 5:**
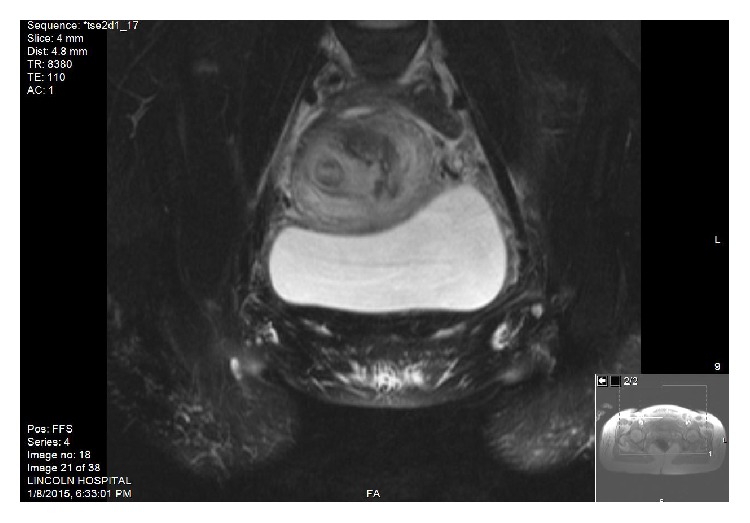
MRI of abdomen and pelvis with contrast: Series # 4 coronal FS (Fast): right uterus and left bulky postpartum uterus.

Pelvic MRI was performed after successful vaginal delivery (Figures 4 and 5) and confirmed this Mullerian duct anomaly to be didelphys uterus which had a longitudinal, complete vaginal septum.

## 3. Discussion

A didelphys uterus remains a very rare Mullerian duct anomaly in comparison to other anomalies described in the Buttram and Gibbons classification. Most of the data on the clinical significance and outcomes of this uterine anomaly are based on small retrospective, observational, or case studies. The results of these studies are mixed, not only due to the types of studies, but also due to the very low incidence of the anomaly in the population and the fact that more research has been directed to the more common malformations: arcuate, septate, bicornuate.

Most women with a didelphys uterus are asymptomatic, but may present with dyspareunia or dysmenorrhea in the presence of a thick, sometimes obstructing, vaginal septum. This obstructing vaginal septum can lead to hematocolpos/hematometrocolpos and thus present as chronic abdominal pain as well. Rarely, genital neoplasms and endometriosis are reported in association with cases of didelphys uterus [1, 2, 14].

The fertility of women with untreated didelphys uterus has been shown by some sources to be better than those with other Mullerian duct abnormalities but still less than women with normal uterine anatomy. There is also an increased risk of spontaneous abortion, fetal growth retardation, and prematurity with an estimated 45% (or lower) chance of carrying a pregnancy to term in comparison to a normal uterus, which is similar to that of a unicornuate uterus. This indicates poor reproductive performance, but still not as poor as a septate or bicornuate uterus which are more common amongst the MDAs [1, 2, 5, 15].

The body of literature on didelphys uterus, although limited, generally shows that the anomaly may lead to better pregnancy outcomes in comparison to the other anomalies; however there are also studies that demonstrate the contrary. For example, Acién's prospective observational study [5] of the reproductive outcome of women with different uterine anomalies in comparison to a normal uterus found the rate of term delivery for a didelphys uterus significantly lower than the normal uterus group but the rate was not as low as that of the bicornuate group and septate group [5]. Grimbizis et al. also confirmed this conclusion in a review on the clinical implications of uterine malformations [4]. Another study by Ludmir et al. also found, with high-risk obstetric intervention, more pregnancies from a didelphys uterus reached term and fetal survival rate was higher in comparison to the bicornuate and septate group [16].

On the other hand, a large retrospective longitudinal study of 3181 patients by Raga et al. demonstrated poor reproductive performance in women with didelphys uteri with a higher rate of preterm delivery, spontaneous abortion, and the lowest chance of having a term delivery than the other MDAs [3]. In addition, a long term retrospective follow-up of 49 women with didelphys uterus found no impairment with fertility and decreased rate of spontaneous abortion; however the rate of prematurity was increased in comparison to other known studies on septate and bicornuate uteri [2].

The association between having a Mullerian duct anomaly and fertility is debatable. The review by Grimbizis demonstrated the incidence of MDAs in infertile patients (3.4%) similar to that of the general population and/or fertile women (4.3%), which they concluded demonstrated that MDAs may not have a negative impact on fertility [4]. To go further, there are reported cases of women with didelphys uteri pregnant with twins or triplets demonstrating the ability to conceive and support the healthy growth of a fetus in either one of the uteri [17–20]. In contrast, the large retrospective study done by Raga et al. found the incidence of Mullerian duct anomalies to be significantly higher in infertile women than in fertile women, suggesting a link between infertility and the MDA [3]. A retrospective study on fertility and obstetric outcome done by Zhang et al. in China demonstrated that women with a didelphys uterus more frequently required infertility treatments than with other anomalies to conceive [21].

Certain procedures may be undertaken to increase fertility, decrease chances of prematurity, and improve the quality of life. Surgical correction of a didelphys uterus (metroplasty) is not usually indicated and the literature on women with didelphys uterus who underwent metroplasty is very limited. With that said, metroplasty would only be considered on a case by case basis after all other ways in which reproductive performance could be improved are exhausted [4, 5, 22]. Observational studies cite women with septate or bicornuate uteri with a history of repeated abortions and infertility demonstrating improvement in reproductive and gestational outcome after metroplasty [4]. Longitudinal vaginal septum excision is considered if the woman is symptomatic, complaining of dyspareunia or pain from hematometrocolpos due to obstruction. Some septa can be easily displaced to the side to facilitate vaginal birth and others may be thick and inelastic, increasing the risks of vaginal dystocia and thus requiring excision. A didelphys uterus is not an indication for cesarean delivery and thus vaginal delivery should be considered first [23–25]. Finally, cervical incompetence is not usually associated with didelphys uterus and thus cerclage is not routinely used unless there is a history of cervical incompetence or premature dilation is found on exam during early second trimester [2, 5, 16].

A didelphys uterus has been shown in many case reports to occur as a part of a syndrome, more specifically called, Herlyn-Werner-Wunderlich (HWW) syndrome, also known as obstructed hemivagina and ipsilateral renal anomaly (OHVIRA). It is a very rare congenital anomaly of the urogenital tract involving Mullerian ducts and Wolffian structures, and it is characterized by the triad of didelphys uterus, obstructed hemivagina, and ipsilateral renal agenesis [26]. This condition can cause hematometrocolpos or hematocolpos on the side of the obstructed hemivagina which produces a mass effect with subsequent lower abdominal pain [8, 27, 28]. Most cases present after menarche as intense lower abdominal pain and/or a protruding mass over the vaginal introitus [8, 27, 28]. Sudden, intense vaginal pain has been documented as a rare presenting symptom as well [28]. A preliminary pelvic Ultrasound is done followed by an MRI to confirm the diagnosis. One case report identified this syndrome in a newborn who was diagnosed with renal agenesis in utero and born with a protruding vaginal mass and a hydrocolpos was found on imaging [29]. Although this condition is extremely rare, it is important for a physician, especially an ER physician, to keep it in mind when a postpubertal female presents with sudden lower abdominal pain and all other causes have been ruled out [8, 27, 28].

## 4. Conclusions

The didelphys uterus is a very rare Mullerian duct anomaly with varying reproductive and gestational outcomes in comparison to other more common abnormalities.

The ability to conceive remains a debatable issue as well. There is insufficient data on surgical correction (metroplasty); therefore it is not usually indicated; however excision of the vaginal septum may be required if the women is symptomatic. Didelphys uterus is not an indication for cesarean delivery unless the vaginal septum is thick and inelastic resulting in an increased risk for vaginal dystocia. Cervical incompetence has not been shown to occur in conjunction with the didelphys uterus. Lastly, when a didelphys uterus is diagnosed, renal anomalies should also be investigated to rule out Herlyn-Werner-Wunderlich (HWW) syndrome.

Overall, the literature available on the didelphys uterus is quite limited at the present time. Therefore more studies are needed in order to better determine the reproductive and gestational outcomes, so that clinicians can adequately advise and care for their patients.
